# Comprehensive Review of In Vitro Human Follicle Development for Fertility Restoration: Recent Achievements, Current Challenges, and Future Optimization Strategies

**DOI:** 10.3390/jcm13061791

**Published:** 2024-03-20

**Authors:** Francisco Vitale, Marie-Madeleine Dolmans

**Affiliations:** 1 Gynecology Research Unit, Institut de Recherche Expérimentale et Clinique, Université Catholique de Louvain, Avenue Mounier 52, 1200 Brussels, Belgium; francisco.vitale@uclouvain.be; 2 Gynecology Department, Cliniques Universitaires Saint-Luc, 1200 Brussels, Belgium

**Keywords:** folliculogenesis, follicle activation, human ovarian tissue, bovine ovarian tissue, in vitro culture, in vitro growth, in vitro maturation

## Abstract

Ovarian tissue cryopreservation (OTC) and subsequent transplantation (OTT) is a fertility preservation technique widely offered to prepubertal girls and young fertile women who need to undergo oncological treatment but are at a high risk of infertility. However, OTT is not considered safe in patients with certain diseases like leukemia, Burkitt’s lymphoma, and ovarian cancer because of the associated risk of malignant cell reintroduction. In vitro follicle development has therefore emerged as a promising means of obtaining mature metaphase II (MII) oocytes from the primordial follicle (PMF) pool contained within cryopreserved ovarian tissue, without the need for transplantation. Despite its significant potential, this novel approach remains highly challenging, as it requires replication of the intricate process of intraovarian folliculogenesis. Recent advances in multi-step in vitro culture (IVC) systems, tailored to the specific needs of each follicle stage, have demonstrated the feasibility of generating mature oocytes (MII) from early-stage human follicles. While significant progress has been made, there is still room for improvement in terms of efficiency and productivity, and a long way to go before this IVC approach can be implemented in a clinical setting. This comprehensive review outlines the most significant improvements in recent years, current limitations, and future optimization strategies.

## 1. Introduction

Young fertile cancer patients undergoing treatment like chemotherapy or radiotherapy face the threat of iatrogenic fertility impairment due to the gonadotoxicity of these interventions [1,2,3,4]. Among fertility preservation techniques developed to address this issue, ovarian tissue cryopreservation (OTC) with subsequent transplantation (OTT) is the only available option for prepubertal girls and patients who cannot postpone their treatment [5,6]. However, auto-transplantation carries the potential risk of reintroducing neoplastic cells, especially in the case of blood-borne malignancies like leukemia and non-Hodgkin’s lymphoma, as well as ovarian cancer [7,8,9].

In recent years, in vitro follicle development has emerged as a promising means of obtaining mature metaphase II (MII) oocytes from the primordial follicle (PMF) pool contained within cryopreserved ovarian cortex, without the need for OTT [10,11]. Indeed, PMFs are a key target for fertility restoration, as they are the most abundant follicle population and can tolerate freezing and thawing procedures [12,13].

Achieving complete in vitro folliculogenesis involves initial activation of quiescent PMFs, further growth and progression through the different stages of development, and then final oocyte maturation prior to in vitro fertilization (IVF). While encouraging, this novel approach remains extremely challenging, since not all signaling mechanisms involved in follicle development are yet fully understood [11,14]. Although it was possible to generate newborns from completely in vitro-derived ovarian follicles in mice well over two decades ago [15], replicating this technique in larger mammals and humans has proven much more problematic. Bovines share greater similarities to humans in terms of reproductive cycles and ovarian follicle kinetics, being a mono-ovular species, in contrast to pigs and mice, which are poly-ovular. Numerous studies were therefore conducted using bovine ovarian tissue as a better model for research into human follicle development.

A few decades ago, insights obtained from ultrasound techniques in cattle paved the way for a deeper understanding of follicle kinetics in humans [16]. Both species’ ovarian cycles exhibit follicle waves characterized by synchronized growth of follicle cohorts, with one eventually emerging as dominant while others regress. During ovulation in both species, LH levels increase, triggering ovulation of the dominant follicle. This shared pattern highlights fundamental parallels in the mechanisms underlying follicle wave generation, dominant follicle selection, and the ultimate release of a single egg in both humans and bovines, making the bovine model an ideal framework for extrapolating results to human clinical applications.

We searched the PubMed database (https://pubmed.ncbi.nlm.nih.gov/, accessed on 1 August 2023) for English-language articles relevant to the subject, published up to June 2023. The search specifically targeted in vitro folliculogenesis studies using the following keywords: ‘in vitro culture’ AND ‘ovarian tissue’ AND ‘ovarian follicles’. A total of 809 articles initially matched these criteria. After identifying original studies that used human or bovine ovarian tissue and were methodologically adequate, the authors selected and reviewed 43 articles. The present review summarizes key advances in in vitro follicle development in humans and bovines in recent years, the main challenges that remain, and strategies to optimize outcomes.

## 2. Different In Vitro Culture Systems

Since the successful creation of mouse embryos from a complete in vitro follicle development system was first reported [15], various research groups have attempted to devise different in vitro culture (IVC) strategies to mimic the same process in humans. Two principal but opposing approaches have been described: isolated PMF culture versus in situ culture of PMFs within ovarian cortex.

Early attempts to culture isolated PMFs failed to induce growth [17,18] and it became clear that preserving interactions between PMFs and neighboring stromal cells in cortical tissue is crucial to their survival and initial growth [19,20,21]. This approach typically involves IVC of thin cortical fragments (no more than 1 mm thick) [10]. Physical factors, including tissue surface area and stiffness, can also compromise in vitro cell behavior. The ovarian cortex must therefore be fragmented and any excess medulla removed to optimize the balance between cultured cells and nutrients. Indeed, disproportion between the tissue surface and medium volume could lead to nutrient insufficiency and tissue necrosis, especially during prolonged IVC [22].

**Figure 1:**
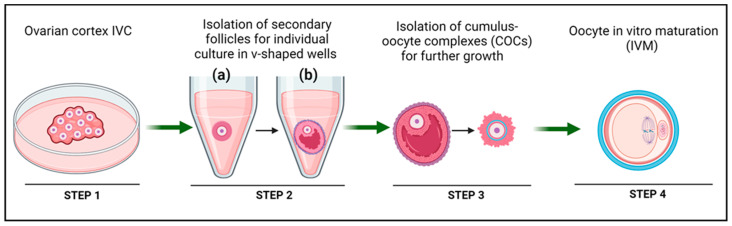
Illustrative representation of the multi-step IVC system supporting in vitro follicle development from PMFs contained within the ovarian cortex to mature MII oocytes, as described by McLaughlin et al., 2018 [10]. Step 1: PMF activation. Step 2: Isolation of secondary follicles (a) and subsequent individual culture in V-shaped wells until the antral follicle stage (b). Step 3: Mechanical dissection of COCs. Step 4: Oocyte IVM until reaching the MII stage. Created with BioRender.com, accessed on 1 January 2024.

Telfer and colleagues recently developed human MII oocytes from PMFs within ovarian tissue using a multi-step IVC procedure [10] (Figure 1). The first step involved activation and early growth of PMFs. Secondary follicles (100–150 µm) were then isolated and individually cultured in V-shaped wells to reach the antral stage, forming cumulus-oocyte complexes (COCs). The final step was maturation of the remaining COCs to the preovulatory stage, enabling collection of mature MII oocytes. 

While this approach served to demonstrate the feasibility of the technique, it also revealed its limited effectiveness, yielding only 9 MII oocytes from 160 initial ovarian tissue fragments. Further refinement of the technique is clearly essential before its implementation in clinical practice. 

## 3. Follicle Activation: From PMFs to Secondary Follicles

Table 1 summarizes different IVC systems, culture periods, medium components, biomaterials, and isolation methods from publications reporting transition of PMFs to secondary follicles in humans and bovines.

### 3.1. Culture Medium Composition for the First Step 

Culture medium composition is pivotal to maintaining viability, growth, and proliferation of cells in an IVC system. The most commonly used basal media for this phase of human ovarian tissue IVC are alpha minimal essential medium (αMEM) [17,19,20,23,24,25,26,27,28,29,30,31,32] and McCoy’s 5a [21,33,34,35,36,37,38,39]. Waymouth’s medium [40,41,42,43,44,45,46] is the most widely used medium for bovine ovarian tissue culture (Table 1). In recent years, there has been a consensus on supplements that should be added to the basal culture medium to support follicle survival and growth. Glucose and amino acids like L-glutamine are usually used as energy sources. Insulin, transferrin, and selenium (ITS) are added to increase uptake of soluble metabolic precursors. Antibiotics such as penicillin and streptomycin are given to prevent microorganism growth. In addition, soluble antioxidants like ascorbic acid are frequently added to culture medium, having been shown to reduce cell apoptosis and increase follicle integrity [47,48,49]. Follicle-stimulating hormone (FSH) and activin A are often included thanks to their effect on granulosa cell (GC) proliferation [10,21]. Medium supplements may also contain serum, such as fetal bovine serum, or serum substitutes, like human serum albumin commonly used as protein complements. 

It is essential to replace culture medium after a fixed amount of time in order to prevent nutrient depletion and ensure elimination of toxic waste products, like ammonia and lactic acid. In studies involving IVC for more than four days, standard practice is to replace half of the culture medium every other day [10,21,38,50,51,52,53].

**Table 1:** Publications reporting transition of PMFs to secondary follicles in humans and bovines (step 1).

Publication.	Source	Type of Culture	Culture Period	Culture Medium	Culture System	Biomaterial	Isolation Method
Hovatta et al., 1997 [19]	Human	Ovarian tissue	21 days	αMEM	2D	Extracellular matrix	N/A
Wright et al., 1999 [20]	Human	Ovarian tissue	15 days	αMEM	2D	Matrigel	N/A
Abir et al., 1999 [17]	Human	Isolated follicles	1 day	αMEM	3D	Collagen gel	Enzymatic
Hreinsson et al., 2002 [24]	Human	Ovarian tissue	14 days	αMEM	2D	Extracellular matrix	N/A
Scott et al., 2004 [25]	Human	Ovarian tissue	7 days	αMEM	N/A	No	N/A
Amorim et al., 2009 [26]	Human	Isolated follicles	7 days	αMEM	3D	Alginate	Enzymatic
Kedem et al., 2011 [54]	Human	Ovarian tissue	14 days	αMEM	3D	Alginate	N/A
Camboni et al., 2013 [27]	Human	Isolated follicles	7 days	αMEM	3D	Alginate	Enzymatic
Lerer-Serfaty et al., 2013 [28]	Human	Ovarian tissue	12 days	αMEM	3D	PEG-fibrinogen	N/A
Wang et al., 2014 [55]	Human	Isolated follicles	8 days	αMEM	3D	Alginate	Enzymatic + mechanical
Laronda et al., 2014 [29]	Human	Isolated follicles	3 days	αMEM	3D	Alginate	Enzymatic
Yin et al., 2016 [56]	Human	Isolated follicles	30 days	αMEM	3D	Alginate	Enzymatic
Hosseini et al., 2017 [30]	Human	Isolated follicles	10 days	αMEM	3D	Alginate	Enzymatic
Hosseini et al., 2019 [57]	Human	Ovarian tissue	8 days	αMEM	N/A	No	N/A
Ghezelayagh et al., 2020 [31]	Human	Ovarian tissue	7 days	αMEM	3D	Agar scaffold	N/A
Ghezelayagh et al., 2021 [32]	Human	Ovarian tissue	7 days	αMEM	3D	Matrigel	N/A
Telfer et al., 2008 [21]	Human	Ovarian tissue	10 days	McCoy’s 5a	N/A	No	Mechanical
Khosravi et al., 2013 [35]	Human	Ovarian tissue	7 days	McCoy’s 5a	N/A	No	N/A
McLaughlin et al., 2011 [33]	Human	Ovarian tissue	6 days	McCoy’s 5a	N/A	No	N/A
McLaughlin et al., 2014 [34]	Human	Ovarian tissue	6 days	McCoy’s 5a	N/A	No	Mechanical
Asadi et al., 2017 [36]	Human	Ovarian tissue	6 days	McCoy’s 5a	N/A	No	N/A
Grosbois et al., 2018 [37]	Human	Ovarian tissue	6 days	McCoy’s 5a	N/A	No	N/A
Hossay et al., 2023 [38]	Human	Ovarian tissue	6 days	McCoy’s 5a	N/A	No	N/A
Subiran Adrados et al., 2023 [39]	Human	Isolated follicles	8 days	McCoy’s 5a	3D	Alginate	Enzymatic + mechanical
Dadashzadeh et al., 2023 [58]	Human	Isolated follicles	7 days	DMEM/F12	3D	PEG hydrogels	Enzymatic
Wandji et al., 1996 [40]	Bovine	Ovarian tissue	7 days	Waymouth	N/A	No	N/A
Fortune et al., 1998 [41]	Bovine	Ovarian tissue	7 days	Waymouth	N/A	No	N/A
Gigli et al., 2006 [42]	Bovine	Ovarian tissue	7 days	Waymouth	N/A	No	N/A
Yang and Fortune, 2006 [43]	Bovine	Ovarian tissue	10 days	Waymouth	N/A	No	N/A
Yang and Fortune, 2007 [44]	Bovine	Ovarian tissue	10 days	Waymouth	N/A	No	N/A
Yang and Fortune, 2008 [45]	Bovine	Ovarian tissue	10 days	Waymouth	N/A	No	N/A
Yang et al., 2017 [46]	Bovine	Ovarian tissue	12 days	Waymouth	N/A	No	N/A

N/A: not applicable.

### 3.2. Molecular Signaling Pathways

Follicle activation is the first step in folliculogenesis and appears to be the key feature of in vitro follicle development. Due to an absence of gonadotropin receptors within PMFs and their limited irrigation supply, PMF activation is most likely gonadotropin-independent, with reliance on paracrine signaling both within follicles and throughout the local intraovarian environment [59]. While still not fully understood, regulation of PMF activation looks to involve intricate coordination between stimulating and inhibiting signals.

#### 3.2.1. Oocyte-GC Crosstalk

Cell communication pathways between the oocyte and GCs are critical features in PMF activation. Previous studies suggest that growth differentiation factor 9 (GDF9) and bone morphogenetic protein 15 (BMP15), two members of the transforming growth factor beta (TGFβ) superfamily specifically secreted by oocytes, may be involved in initiating follicle growth and subsequent stage transition [60,61]. It has been documented that after follicle activation, the recruited oocyte initiates GDF9 and BMP15 secretion, directly impacting both GC proliferation and expansion, and thereby promoting follicle transition [62,63]. Indeed, addition of human recombinant GDF9 and BMP15 to human ovarian tissue IVC has been found to promote increased activation of PMFs and higher estradiol secretion [54]. On the other hand, GDF9 knockout mice showed significant impairment of follicle development, which hampers progression beyond the primary stage [64], while BMP15 knockout mice exhibited subfertility, with lower ovulation and fertilization rates [65]. 

Another hormone regulating follicle activation is anti-Müllerian hormone (AMH) [66], also a member of the TGFβ superfamily. AMH is secreted by GCs from the primary follicle stage onwards and peaks during the secondary and small antral follicle stages [67]. AMH from growing follicles has an inhibitory effect on follicle activation in neighboring quiescent PMFs [68], designed to maintain a balanced and coordinated process of follicle recruitment and development. Certainly, studies in AMH-knockout mice revealed increased numbers of antral follicles, coupled with a decrease in the PMF count [69]. It has also been demonstrated that supplementing human [70] and bovine [46] ovarian tissue IVC with AMH curbs follicle activation.

#### 3.2.2. Hippo Signaling

**Figure 2:**
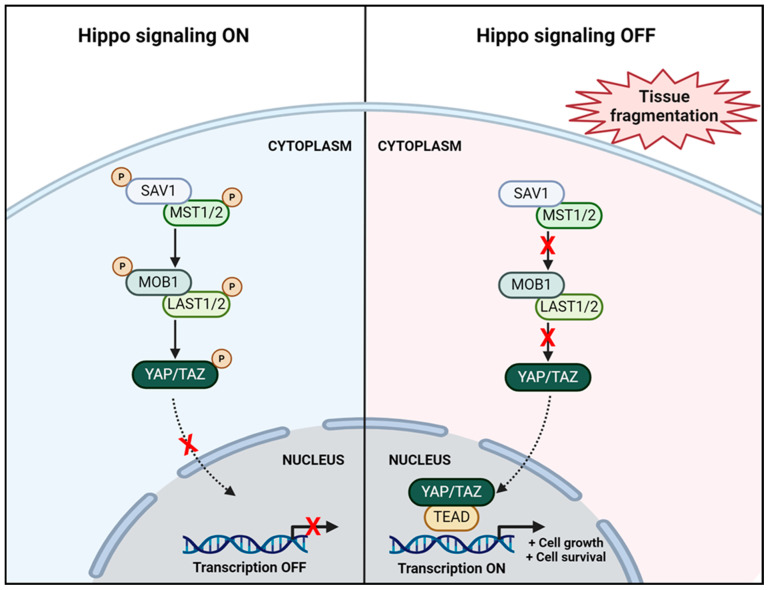
When the Hippo pathway is active (**left**), SAV1 and MST1/2 complex phosphorylates LATS1/2 and MOB1. Activated LATS1/2 subsequently phosphorylates the YAP/TAZ complex, resulting in cytoplasmic retention and no DNA transcription. Conversely, when the Hippo pathway is disrupted (**right**) during ovarian tissue fragmentation, dephosphorylated YAP1/TAZ translocates to the nucleus to bind with TEAD, leading to transcriptional activation of genes associated with cell growth and survival. Created with BioRender.com. Abbreviations: LATS1/2 (large tumor suppressor kinase 1/2); MOB1 (Mps one binder 1); MST1/2 (mammalian Ste20-like serine/threonine kinases 1/2); P (phosphorylated); SAV1 (protein salvador homolog 1; TEAD (TEA domain family members).

Among the different molecular pathways, Hippo signaling appears to play a key role in PMF activation (Figure 2). This pathway regulates organ size, tissue homeostasis, and cell differentiation [71]. The Hippo pathway functions through downstream effectors, namely transcriptional coactivator yes-associated protein (YAP) and transcriptional coactivator PDZ-binding motif (TAZ) [72,73]. While active, this kinase-regulated suppressive pathway eventually causes phosphorylation of the YAP/TAZ complex, resulting in its retention and degradation within the cytoplasm, and thereby preventing its nuclear localization and activation of transcription factors. Conversely, during ovarian tissue fragmentation, transformation of globular actin into filamentous actin disrupts this signaling pathway, leading to accumulation of unphosphorylated YAP/TAZ in the nucleus, which subsequently enhances cell proliferation-related gene expression [72,74,75,76]. Lunding and colleagues demonstrated that fragmentation of human ovaries boosted actin polymerization, causing inhibition of the Hippo pathway by dephosphorylation and nuclear translocation of YAP, and ultimately leading to follicle and oocyte growth [77]. Likewise, immunostaining techniques (targeting YAP) on human ovarian tissue have revealed that in vitro tissue fragmentation activates PMFs through the Hippo pathway [78]. Grosbois and colleagues were even able to prove that after IVC, follicles situated closer to the fragmentation site were more developed than those localized deeper in cortical tissue [37].

#### 3.2.3. PI3K/AKT Pathway

**Figure 3:**
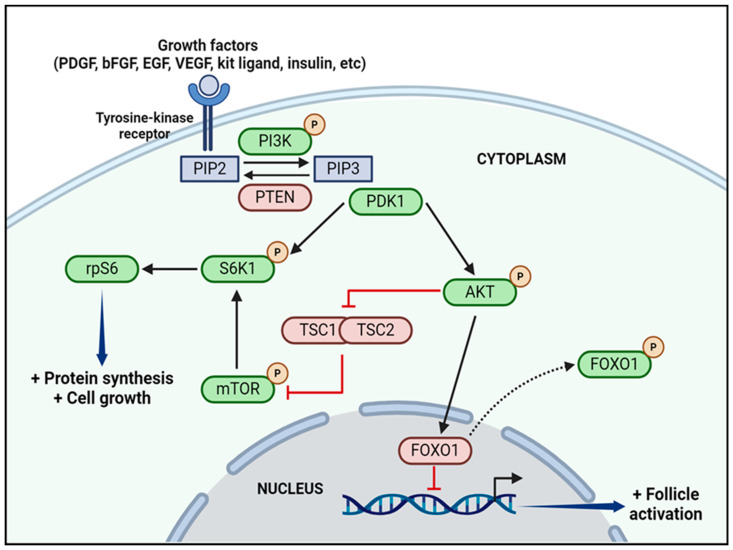
The PI3K/AKT pathway is activated following binding of several growth factors to tyrosine-kinase receptors on cell membranes. This interaction leads to PIP2 transformation into PIP3. AKT is then phosphorylated and translocated to the nucleus where it phosphorylates FOXO1, resulting in its export into the cytoplasm. After translocation, inactive FOXO1 ceases its inhibitory effect over transcriptional factors, enhancing follicle activation and growth. mTOR, another AKT downstream effector regulates protein synthesis and cell growth through ribosomal biosynthesis, also promoting follicle activation. PTEN, on the other hand, counteracts the conversion of PIP2 into PIP3, inhibiting the pathway. Activators are represented in green and inhibitors in red. Created with BioRender.com. Abbreviations: AKT (protein kinase B); bFGF (basic fibroblast growth factor); EGF (epidermal growth factor); FOXO1 (forkhead box O1); mTOR (mammalian target of rapamycin); P (phosphorylated); PDGF (platelet-derived growth factor); PDK1 (phosphoinositide-dependent kinase-1); PI3K (phosphatidylinositol 3-kinase); PIP2 (phosphatidylinositol 4,5-bisphosphate); PIP3 (phosphatidylinositol 3,4,5-trisphosphate); PTEN (phosphatase and tensin homolog); rpS6 (ribosomal protein S6); S6K1 (S6 kinase 1); TSC1 and TSC2 (tuberous sclerosis complex 1 and 2); VEGF (vascular endothelial growth factor).

The phosphoinositide 3-kinase (PI3K)-protein kinase B (AKT) signaling pathway has also been implicated in PMF activation [34,50] (Figure 3). The PI3K/AKT pathway is activated by various growth factors. Platelet-derived growth factor (PDGF) and basic fibroblast growth factor (bFGF) are among those able to trigger oocyte activation by boosting oocyte-GC crosstalk through c-kit/kit ligand signaling [59,79,80]. Upon binding of the c-kit receptor to the oocyte membrane, increased kit ligand expression and secretion from GCs activate the PI3K/AKT pathway [81,82]. Other growth factors such as vascular endothelial growth factor (VEGF), epidermal growth factor (EGF), and hormones like insulin are able to directly stimulate the PI3K/AKT pathway upon binding to tyrosine-kinase receptors [59,79,83]. After receptor activation, phosphatidylinositol-4,5-bisphosphate (PIP2) phosphorylates into phosphatidylinositol 3,4,5-triphosphate (PIP3). AKT is then phosphorylated and translocated to the nucleus, where it in turn phosphorylates the transcriptional factor forkhead box O (FOXO), resulting in its export into the cytoplasm. After translocation, inactive FOXO ceases its inhibitory influence over follicle growth [84,85]. Mammalian target of rapamycin complex (mTORC), another AKT downstream effector, is also involved in early-stage follicle activation and development. When active, mTORC regulates protein synthesis and cell growth through ribosomal biogenesis, enhancing follicle activation [86]. Conversely, phosphatase and tensin homolog (PTEN) has a negative impact on PI3K/AKT signaling, counteracting conversion of PIP2 into PIP3 [75]. Past research has demonstrated upregulation of the PI3K/AKT and mTORC pathways and a decrease in PTEN signaling upon analysis of oocyte transcriptomic profiles during primordial-to-primary follicle transition in human ovarian follicles [87]. 

### 3.3. Spontaneous In Vitro Follicle Activation: Friend or Foe? 

In vivo, PMF quiescence is maintained by an intricate balance between stimulatory and inhibitory autocrine and paracrine cues within the intraovarian setting. However, follicle activation occurs spontaneously in vitro after a few days in both humans [10,21,38,50] and bovines [40,41]. This might be due to disruption of follicle activation-suppressing mechanisms after the cortical fragment is extracted from its natural environment [88]. Such uncontrolled in vitro activation stands in sharp contrast to the natural physiological process, where PMFs are gradually recruited in regulated waves. Indeed, this highly coordinated development is estimated to take at least 80 days in vivo [89], raising questions about the quality and genomic integrity of in vitro-derived follicles that reach the same growth stage in around 10 days. Previous studies [10,21] have in fact found that despite rapid in vitro activation, only a limited number of PMFs are capable of progressing to the next stage of follicle growth, while the majority face follicle death or development arrest. Not all activated follicles manage to grow and develop to further stages [10], but whether this developmental defect lies in the initial uncontrolled activation or happens at some later stage cannot yet be determined. 

In recent years, numerous investigations have sought to increase follicle activation using pharmacological agents to enhance ovarian tissue IVC. Short-term in vitro exposure to low doses of PTEN inhibitors like bisperoxovanadium(pic) [bpV(pic)] or bisperoxovanadium(HOpic) [bpV(HOpic)] was found to improve human PMF activation and growth in vitro [34,90] and promote estradiol secretion [90]. Creating a favorable environment for follicle growth could certainly be beneficial in clinical settings. Kawamura and colleagues reported human pregnancies after grafting ovarian cortex previously exposed to a PTEN inhibitor to patients with premature ovarian failure [72,91,92]. However, iatrogenically forcing follicle activation may not be harmless to follicle health. Apart from its function in follicle activation, PTEN also plays a role in maintaining genomic stability [93,94]. Indeed, studies have demonstrated that PTEN inhibition causes greater follicle DNA damage, impairs DNA repair mechanisms [95] and increases histomorphological follicle abnormalities, such as loss of GC-oocyte contacts, steroidogenesis defects, and poor survival of growing follicles [28,34,37]. 

Conversely, other researchers have hypothesized that an ideal IVC system should limit extensive follicle activation to mimic the natural intraovarian environment. Pharmacological inhibition of mTORC, a downstream effector of the PI3K/AKT pathway, has been used to attenuate follicle in vitro activation. Exposure to rapamycin, an mTORC1 inhibitor, resulted in high rates of oocyte loss and an ‘empty follicle’ pattern in ovarian tissue culture [33]. Surprisingly, better outcomes were observed with everolimus (EVE), an analog of rapamycin. EVE has been reported to have a protective effect on maintaining PMF dormancy and avoiding IVC-induced spontaneous activation [37]. Furthermore, adding AMH to ovarian tissue IVC could be a valuable approach to control follicle activation. Recombinant AMH exposure was also shown to prevent PMF activation in cultured ovarian tissue both in humans [70] and bovines [46].

Regulation of in vitro activation by ovarian tissue IVC offers a promising avenue for fertility treatments but raises concerns about follicle health and genetic integrity. While the results may look encouraging, it is essential to remain cautious regarding potential impairments to follicle health, quality, and genetic and epigenetic integrity. Long-term impacts of genetic instability on oocytes and subsequent offspring remain uncertain. Indeed, these new reproductive techniques still have a long way to go before they can be safely employed in a clinical setting [96].

### 3.4. Mimicking the In Vivo Environment: The Key to Success

Physical and biological parameters like base media and additives, nutrients, temperature, oxygen (O_2_) tension, and light exposure should be meticulously analyzed to determine the optimal IVC strategy. Ultimately, the IVC system that most closely mimics the intraovarian physiological environment is one that causes the least cell distress and yields the best viability.

Optimal temperatures for IVC can vary from species to species depending on normal body temperatures, like 37 °C for human tissue and 38.5–39 °C for bovine tissue. Determining species-specific temperature requirements is crucial to successful IVC.

In recent years, cell-based co-culture systems have attracted attention for their potential to replicate the intraovarian microenvironment [97,98]. It appears that ‘feeder cells’, such as different types of mesenchymal stem cells (MSCs), exert their influence on neighboring cells due to their capacity to release a secretome containing cytokines, chemokines, and growth factors. Among these cells, bone marrow-derived (BM)-MSCs were found to enhance follicle growth and decrease follicle apoptosis in a human ovarian tissue co-culture model [57]. It has also been very recently demonstrated that addition of adipose tissue-derived stem cell (ASC)-conditioned medium, which includes the secretome, to bovine ovarian tissue IVC significantly boosts follicle viability, development, and estradiol secretion [99]. Indeed, MSC derivatives like conditioned medium could emerge as powerful optimization tools, as they reduce the risk of cell differentiation and nutrient competition within shared culture media. They also provide a more secure option, given its ease of collection, storage, and standardization, thereby ensuring consistent and reproducible outcomes.

Oxygen tension is another crucial environmental factor affecting IVC follicle outcomes. Optimal O_2_ tension is difficult to determine in culture. It is estimated that quiescent PMFs reside within the ovarian cortex at physiological O_2_ tension levels ranging between 2% and 8% [100,101]. Elevated O_2_ tension causes accumulation of reactive oxygen species (ROS), eventually leading to oxidative stress damage and cell dysfunction. Consequently, culturing PMFs at O_2_ tension beyond physiological levels may result in increased follicle distress and reduced viability. In line with these data, a study reported that human ovarian tissue cultured at 5% O_2_ tension yielded lower follicle apoptosis rates, mainly by generating less oxidative stress damage and fewer DNA double-strand breaks [51] than culture at 20% O_2_ tension. It was also reported that hypoxia induces a dormant state in oocytes through FOXO3, a downstream effector of the PI3K/AKT signaling pathway [102]. Atmospheric O_2_ tension could therefore be another factor contributing to large-scale spontaneous human follicle activation invariably observed in vitro. 

Conversely, there is no clear directive on O_2_ tension in bovine ovarian tissue IVC. Jorssen and colleagues found no significant differences in follicle viability or growth between 5% and 20% O_2_ tension [103]. Although the role of O_2_ tension has not yet been fully elucidated, it is clear that it varies according to follicle stage. Low O_2_ tension is most critical during the early stages of IVC, while higher tension may be required during later stages to support normal development of GCs and steroidogenesis [42]. This mirrors in vivo follicle dynamics, where PMFs migrate from the avascular periphery towards the highly irrigated medulla as they grow.

Finally, it is worth noting that low in vitro survival and growing follicle rates are likely due to suboptimal culture medium composition. Determining which culture supplements should be added to enhance IVC outcomes is extremely challenging, as factors, proteins, and signaling pathways involved in follicle activation, growth, and maturation are still largely unknown. This lack of understanding of the complex processes of folliculogenesis is undoubtedly a significant limitation to achieving favorable in vitro follicle outcomes.

## 4. Follicle In Vitro Growth (IVG): From Secondary to Antral Follicles

Table 2 summarizes different IVC systems, culture periods, medium components, biomaterials, and isolation methods from publications reporting transition of secondary follicles to antral follicles in humans and bovines.

### 4.1. Isolation Techniques 

After reaching the secondary follicle stage consisting of a multilayer of GCs, follicles cannot survive within the cortical environment, so isolation from surrounding ovarian cells is a prerequisite for further in vitro development. This is not surprising as, during intraovarian development, follicles migrate from the rigid cortex towards the less dense medulla. Secondary follicle isolation can be performed either enzymatically [56,58,104,111], mechanically by microdissection [10,21,23,34,52,53,107,109,110], or a combination of both [39,105,106] (Table 2). The microdissection approach, using fine-gauge needles, has been established as the most appropriate, as it maintains an intact follicle basement membrane, thereby preserving oocyte-GC communications [10,21].

### 4.2. Secondary Follicle Culture Systems and Medium Composition

The physical setting of isolated follicles is hugely important at this stage. In the past, follicle IVG studies only took a few days, and 2D culture systems enabling follicles to attach to a flat surface appeared to function adequately [104]. However, with establishment of long-term IVC techniques, the 2D method exhibited significant limitations, such as loss of cell-to-cell communication and follicle growth arrest [112]. Researchers, therefore, shifted to 3D culture systems using biomaterials to encapsulate follicles to better mimic the intraovarian environment (Table 2). Among these bio-matrices, natural compounds like alginate and Matrigel (a commercialized solubilized basal membrane matrix) were found to support IVG of isolated human secondary follicles [56,105]. Moreover, synthetic components such as polyethylene glycol (PEG)-ylated fibrin hydrogels were successfully utilized to promote human secondary follicle development in vitro [58]. Other approaches using decellularized ovarian tissue [113] and 3D microporous scaffolds [114] were also shown to support follicle IVG. However, IVG can in fact be performed without any extracellular matrix or scaffold at all [10]. In the multi-step IVC system, isolated secondary follicles can be cultured individually in V-shaped well culture dishes without any added biomaterial until the antral stage [10]. Numerous studies on isolated bovine follicles have consistently demonstrated that V-shaped microwell plates facilitate follicle growth and proliferation [52,109,110], although the droplet culture approach has also been successfully applied [53]. 

Medium composition is also crucial to IVG. The most commonly used media in human IVG systems are αMEM [23,56,105,107,108], McCoy’s 5a [10,21,34,39], Dulbecco’s MEM (DMEM) [104], or mixed media (DMEM+F12) [58], while McCoys’ 5a [109,110] and TCM-199 [53] are typically utilized in bovines (Table 2). 

Addition of activin A and low-dose FSH at this stage has been found to impart a stabilizing influence on intercellular connections, improve the quality of oocytes and promote antrum formation in both humans [10,21,107] and bovines [115]. In this context, it has been reported that FSH receptors (FSHRs) are mainly present during growth stages [116] and, upon binding to FSH, they initiate intracellular mechanisms involved in GC proliferation [117]. Activin has also been shown to act in coordination with FSH, preserving the integrity of intercellular connections within follicles [118]. In oocytes, activin is involved in modulation of nuclear gene transcription, promoting maturation [119,120]. Isolated follicle growth and survival can also be enhanced with other culture additives such as bFGF [55], antioxidants like ascorbic acid that mitigate oxidative stress damage [21,47], and platelet-rich plasma or human platelet lysate containing high concentrations of growth factors [30,39]. While various research groups have tested different additives, further studies are needed to determine their effectiveness. It is crucial to establish clear guidelines on exactly which supplements should be added to standard IVC growth medium. 

### 4.3. Antrum Formation

This step involves enlargement of the oocyte, further replication and expansion of GCs, and formation of a central fluid-filled cavity known as the antrum. As preantral follicles develop, areas of fluid initially accumulate between GCs, eventually leading to the creation of a large central antrum. This central fluid-filled space serves as a reservoir for various substances and plays a crucial role in providing essential support and nourishment to the oocyte as it continues to mature. Based on in vivo migration of growing follicles from the cortex to the medulla during physiological development, it is thought that antrum formation and expansion might be influenced by biomechanical environmental factors. Follicles in collagen-dense ovarian cortex are less likely to grow, while those in the medulla benefit from a biomechanical environment that supports further development and antrum formation [121]. Xiao and colleagues found that while human follicles encapsulated in alginate could grow to a diameter of 110 μm after 30 days, oocytes within these follicles were unable to progress to the MII stage, instead remaining at the germinal vesicle (GV) stage or deteriorating, most probably due to limitations imposed by the physical surroundings. However, when antral follicles were removed from the alginate hydrogel and further cultured in low-attachment plates using a dynamic two-step system, they were able to reach the MII stage [107]. Indeed, these findings emphasize the importance of providing a dynamic in vitro environment for follicle development.

## 5. Oocyte In Vitro Maturation (IVM): The Final Step

**Figure 4:**
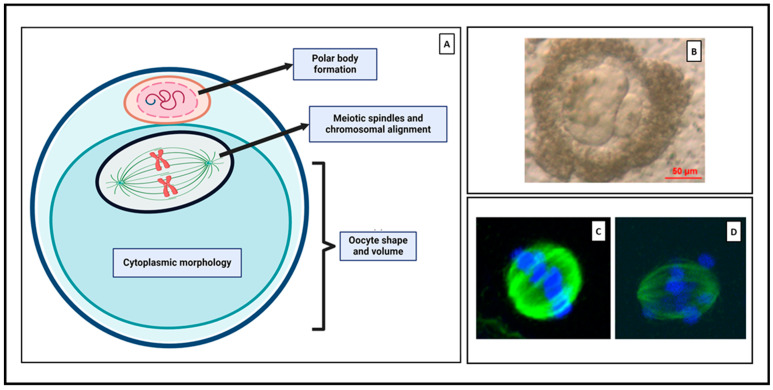
(**A**) Schematic representation of morphological parameters used to assess oocyte competence. Created with BioRender.com. (**B**) Bright field image showing a MII oocyte with and enlarged abnormal polar body. Reproduced with permission from [10]. Confocal images displaying (**C**) equatorially aligned chromosomes (blue) and meiotic spindles (green), and (**D**) chromosomal misalignment. Reproduced with permission from [128].

This technique has advanced significantly over the last 30 years [122,123,124]. It involves oocyte maturation to achieve meiosis resumption, chromatin condensation, development of the meiotic spindle, and expulsion of the initial polar body, reaching the mature stage of MII oocyte [125] (Figure 4A). This technique can be performed either on (i) immature oocytes obtained from oocyte pick-up or (ii) oocytes from in vitro-derived follicles. The former technique entails puncturing small and mid-antral follicles before they reach periovulatory size ranges (between 6–12 mm) without previous hormone stimulation, and final oocyte maturation is achieved in vitro [123]. In this review, our focus is on the latter option, where IVM is performed on completely in vitro-derived follicles. Table 3 summarizes different IVC systems, culture periods, medium components, and use of biomaterials from publications reporting oocyte IVM from in vitro-derived follicles.

### IVM from In Vitro-Derived Follicles 

Achieving successful IVM from in vitro-derived follicles poses considerable technical challenges. Oocyte competence is gained progressively throughout follicle development and involves gradual accumulation of RNA molecules and proteins throughout oocyte growth, which will constitute the oocyte genome [79]. Previous research in mammals has shown that the oocyte genome may be strongly influenced by the environment [126], so follicle IVC will clearly have an impact on oocyte RNA and protein regulation. Environmental epigenomic modifications mainly include DNA methylation, chromatin reorganization, and histone modifications [127], all of which contribute to proper segregation of chromosomes during meiosis.

To date, only three research groups have documented successful maturation of oocytes from cultured human follicles (Table 3), albeit invariably showing suboptimal oocyte developmental competence. Unlike animal models where fertilization rates can be measured, the only viable options to assess human oocyte developmental competence are morphological parameters like establishment of the meiotic spindle, chromosomal alignment, polar body formation, and cytoplasmic ultrastructure, obviously due to ethical concerns (Figure 4A–D).

Xiao and colleagues reported the generation of MII oocytes from mechanically isolated secondary follicles, with a typical meiosis spindle configuration. However, polar body fragmentation was observed [107], which denotes low oocyte quality for potential IVF. Likewise, McLaughlin’s team achieved MII oocyte production from early-stage follicles cultured in a multi-step IVC system, but this approach exhibited limited effectiveness, yielding only 9 MII oocytes from 160 ovarian tissue fragments. Furthermore, these oocytes displayed abnormally large polar bodies [10]. Similarly, Xu’s team demonstrated development of MII oocytes from early-stage follicles cultured in situ within cortical fragments in a more recent study. By the end, 3 out of 14 MII oocytes showed normal spindle configuration, adequate polar body size, and typical intracellular ultrastructure [108]. All in all, these results highlight the challenges associated with achieving optimal oocyte competence using in vitro folliculogenesis systems. 

## 6. Future Directions

Creating a successful and efficient long-term IVC system for human follicles is a demanding pursuit. Researchers have recently been exploring ways of generating dynamic microfluidic culture systems in assisted reproduction devices, such as reproductive organs-on-a-chip [129], in vitro spermatogenesis [130], and testis culture [131]. The dynamic microfluidic approach aims to establish a constant flow of culture medium around tissue, closely mimicking the physiological ovarian microenvironment by facilitating continuous exchange of metabolites and cell waste. This innovative technique might have the potential to overcome limitations associated with static IVC approaches, hopefully improving follicle survival and development. Moreover, employing a dynamic O_2_ tension IVC system could be advantageous. As previously mentioned, quiescent PMFs initially reside in the avascular cortical region and gradually migrate to the highly irrigated medulla, as they progress through developmental stages [89]. This increasing O_2_ gradient is crucial to GC proliferation, steroidogenesis, and oocyte maturation during follicle growth [132]. Indeed, applying dynamic O_2_ tension throughout IVC could positively impact follicle quality and competence, as it mimics O_2_ gradients experienced during physiological ovarian follicle development.

Another avenue for advancement involves implementation of ovarian organoids. This concept refers to in vitro generation of miniature histological structures resembling ovarian source tissue [133]. Such a 3D approach could be used not only as an alternative for fertility restoration purposes, but also as a novel opportunity to investigate disease mechanisms, the impact of gonadotoxic agents, and potential therapeutic strategies. Li and colleagues recently developed an ovarian organoid using mouse female germline stem cells, resulting in differentiated heterogenic tissue containing germ cells and somatic cells like GCs and theca cells [134]. This model demonstrated reproductive functions, including oocyte and offspring production, and endocrine activity, with secretion of progesterone and estradiol. Use of ovarian organoids in humans certainly holds great promise as a brand-new approach to fertility restoration, offering the potential for improved outcomes and broader applications in the field of reproductive medicine.

To validate such innovative strategies for human tissue in clinical practice, it is imperative to follow a structured approach. The European Society of Human Reproduction and Embryology (ESHRE) task force on ethics and law outlines a comprehensive research pathway to evaluate the efficacy and safety of new assisted reproductive technologies, including four key steps: (i) conducting animal studies; (ii) undertaking preclinical embryo research; (iii) performing clinical trials on human subjects; and (iv) conducting follow-up studies to monitor long-term outcomes [135]. Research in animals has already been developed according to these principles, but perhaps the most challenging step is human embryo research, because of ethical regulations that restrict this practice in many European countries. That said, it is also very important to stress that regulatory considerations, including obtaining ethical approval and compliance with medical device regulations from institutional boards, are crucial throughout the entire validation process. This systematic approach should be adhered to, so that approval for these innovative techniques can be granted before their integration into clinical settings.

## 7. Patient Perspectives

A diagnosis of cancer represents a profound challenge for young women, triggering both the psychological shock of the diagnosis itself and the potential repercussion on fertility due to gonadotoxic treatments [136]. Concerns about fertility can indeed dash their hopes of a family, causing considerable emotional distress. Moreover, facing the complex landscape of treatment decisions and medical interventions associated with cancer therapy can be hugely overwhelming. Among oncological patients, those with pathologies that contraindicate OTT are most acutely affected by this uncertainty. For this category of patients, we may advocate OTC for fertility preservation in very young girls, in the hope of continued (albeit slow and difficult) progress in the field in the future.

## 8. Conclusions

In vitro follicle development has shown significant potential as a novel method for fertility restoration in young cancer patients with OTT contraindications. Despite ongoing challenges associated with the in vitro technique, some studies have demonstrated the ability to generate mature human MII oocytes from early-stage follicles. Indeed, there is a widely accepted consensus on the benefits of culturing PMFs in situ within ovarian cortex to achieve follicle activation, along with mechanical microdissection of secondary follicles for further growth. In addition, IVC medium composition has been standardized over the years, albeit with slight variations between species, the most common being αMEM and McCoy’s 5a for humans and Waymouth and TCM-199 for bovines. 

New strategies, such as dynamic microfluidic culture systems and dynamic O_2_ tension IVC systems, aim to better replicate the physiological ovarian microenvironment, potentially enhancing follicle survival and development rates. The use of ovarian organoids offers exciting prospects for both fertility restoration and investigation of disease mechanisms and therapeutic strategies. 

Further optimization and refinement could ultimately make in vitro follicle development a safe, accessible, and cost-effective option for fertility restoration in a clinical setting, providing a valuable alternative for subjects who cannot undergo OTT. 

## Figures and Tables

**Table 2:** Publications reporting transition of secondary to antral follicles in humans and bovines (step 2).

Publication	Source	Culture Period	Culture Medium	Culture System	Biomaterial	Isolation Method
Roy et al., 1993 [104]	Human	5 days	D-MEM	2D	Agar	Enzymatic
Abir et al., 1997 [23]	Human	28 days	αMEM	2D	Extracellular matrix	Mechanical
Xu et al., 2009 [105]	Human	30 days	αMEM	3D	Alginate	Enzymatic + mechanical
Xia et al., 2015 [106]	Human	8 days	αMEM	3D	Alginate	Enzymatic + mechanical
Xiao et al., 2015 [107]	Human	40 days	αMEM	3D	Alginate	Mechanical
Yin et al., 2016 [56]	Human	30 days	αMEM	3D	Alginate	Enzymatic
Telfer et al., 2008 [21]	Human	10 days	McCoy’s 5a	V-shaped microwell	No	Mechanical
McLaughlin et al., 2014 [34]	Human	6 days	McCoy’s 5a	V-shaped microwell	No	Mechanical
McLaughlin et al., 2018 [10]	Human	23 days	McCoy’s 5a	V-shaped microwell	No	Mechanical
Xu et al., 2021 [108]	Human	42 days	αMEM	N/A	No	Mechanical
Subiran Adrados et al., 2023 [39]	Human	8 days	McCoy’s 5a	3D	Alginate	Enzymatic + mechanical
Thomas et al., 2007 [109]	Bovine	6 days	McCoy’s 5a	V-shaped microwell	No	Mechanical
McLaughlin and Telfer, 2010 [110]	Bovine	15 days	McCoy’s 5a	V-shaped microwell	No	Mechanical
Rossetto et al., 2013 [52]	Bovine	16 days	α-MEM, McCoy’s 5a and TCM-199	V-shaped microwell	No	Mechanical
Paulino et al., 2018 [53]	Bovine	18 days	TCM-199	Droplets culture	No	Mechanical

N/A: not applicable.

**Table 3:** Publications reporting the generation of mature oocytes MII from in vitro-derived human follicles.

Publication	Source	Type of Culture	Culture Period	Culture Medium	Culture System	Biomaterial
Xiao et al., 2015 [107]	Human	Isolated follicles	40 days	αMEM	3D	Alginate
McLaughlin et al., 2018 [10]	Human	Ovarian tissue	23 days	McCoy’s 5a	N/A	No
Xu et al., 2021 [108]	Human	Ovarian tissue	42 days	αMEM	N/A	No

N/A: not applicable.

## Data Availability

No new data were created or analyzed in this study. Data sharing is not applicable to this article.
